# Minimizing acetate formation from overflow metabolism in *Escherichia coli*: comparison of genetic engineering strategies to improve robustness toward sugar gradients in large-scale fermentation processes

**DOI:** 10.3389/fbioe.2024.1339054

**Published:** 2024-02-14

**Authors:** Greta Gecse, Rugile Labunskaite, Margit Pedersen, Mogens Kilstrup, Ted Johanson

**Affiliations:** ^1^ dsm-firmenich, Hørsholm, Denmark; ^2^ Department of Biotechnology and Biomedicine, Technical University of Denmark, Kgs. Lyngby, Denmark

**Keywords:** *Escherichia coli*, 2’-*O*-fucosyllactose, acetate, overflow metabolism, genetic engineering, large-scale fermentation, tricarboxylic acid cycle, phosphotransferase system

## Abstract

**Introduction:** *Escherichia coli*, a well characterized workhorse in biotechnology, has been used to produce many recombinant proteins and metabolites, but have a major drawback in its tendency to revert to overflow metabolism. This phenomenon occurs when excess sugar triggers the production of mainly acetate under aerobic conditions, a detrimental by-product that reduces carbon efficiency, increases cell maintenance, and ultimately inhibits growth. Although this can be prevented by controlled feeding of the sugar carbon source to limit its availability, gradients in commercial-scale bioreactors can still induce it in otherwise carbon-limited cells. While the underlying mechanisms have been extensively studied, these have mostly used non-limited cultures. In contrast, industrial production typically employs carbon-limited processes, which results in a substantially different cell physiology.

**Objective:** The objective of this study was to evaluate and compare the efficiency of different metabolic engineering strategies with the aim to reduce overflow metabolism and increase the robustness of an industrial 2’-*O*-fucosyllactose producing strain under industrially relevant conditions.

**Methods:** Three distinct metabolic engineering strategies were compared: i) alterations to pathways leading to and from acetate, ii) increased flux towards the tricarboxylic acid (TCA) cycle, and iii) reduced glucose uptake rate. The engineered strains were evaluated for growth, acetate formation, and product yield under non-limiting batch conditions, carbon limited fed-batch conditions, and after a glucose pulse in fed-batch mode.

**Results and Discussion:** The findings demonstrated that blockage of the major acetate production pathways by deletion of the *pta* and *poxB* genes or increased carbon flux into the TCA cycle by overexpression of the *gltA* and deletion of the *iclR* genes, were efficient ways to reduce acetate accumulation. Surprisingly, a reduced glucose uptake rate did not reduce acetate formation despite it having previously been shown as a very effective strategy. Interestingly, overexpression of *gltA* was the most efficient way to reduce acetate accumulation in non-limited cultures, whereas disruption of the *poxB* and *pta* genes was more effective for carbon-limited cultures exposed to a sudden glucose shock. Strains from both strategies showed increased tolerance towards a glucose pulse during carbon-limited growth indicating feasible ways to engineer industrial *E. coli* strains with enhanced robustness.

## 1 Introduction

Microbial cell factories have become a vital tool for producing a wide range of valuable compounds, including recombinant proteins, bulk and fuel chemicals, pharmaceuticals, medical foods and dietary supplements. One such example is human milk oligosaccharides (HMOs): a family of structurally diverse unconjugated glycans that, after lactose and fat, collectively compose the third largest component of mothers’ milk (Bode, 2012). HMOs are uniquely diverse and abundant in human milk compared to that of domesticated animals (Urashima et al., 2013) and are of particular interest due to their beneficial impact on infants’ and adults’ health. Clinical and preclinical data demonstrate that HMOs support gastrointestinal health, immune function and cognitive development (recently reviewed by Hill May 2021; Sprenger et al., 2022; Dinleyici et al., 2023). They have therefore been introduced into both infant formula as well as into dietary supplementation products for adults (Bych et al., 2019; Lu, Mosleh, and Abbaspourrad, 2021; Pérez-Escalante et al., 2022). The most abundant HMO in human milk, 2’-*O*-fucosyllactose (2’FL), is currently produced on a commercial scale by fermentation using predominantly *E. coli* as production host (Ammann, 2017; Vandenplas et al., 2018; Bych et al., 2019). These fermentations are fully aerobic and employ a fed-batch feeding strategy with carbon limitation to avoid overflow metabolism, reduce the oxygen transfer rate demand and to maximize the carbon source utilization (using either glucose, sucrose or glycerol as carbon source) (Krause, Neubauer, and Neubauer, 2016). Unlike batch growth, where cells have unlimited carbon source availability, a fed-batch regulates the availability such that the respiratory metabolism of the cells and oxygen transfer rate capacity of the process are not overwhelmed (Enfors and Häggström, 1999; Srivastava and Gupta, 2011). This will otherwise result in overflow metabolism or anaerobic metabolism and the formation of undesirable metabolic by-products, predominantly in the form of acetate in *E. coli* (Krause et al., 2016; Millard et al., 2021; Li et al., 2022). Not only does acetate production constitute a wasteful use of the carbon source, but acetate also has a toxic effect that hinders growth (Pinhal et al., 2019), decreases the stability of intracellular proteins and limits product yields and titers (Eiteman and Altman, 2006; De Mey, De Maeseneire, et al., 2007). Such impacts have been observed in *E. coli*, even at relatively low acetate concentrations (Roe et al., 2002; Warnecke and Gill, 2005). Despite using a carbon-limited mode of operation during manufacturing, several circumstances in the industrial setting can lead to exposure to elevated sugar concentrations and induce overflow metabolism. The longer mixing times encountered in large-scale commercial vessels lead to the formation of local zones with significantly elevated concentrations around the feed inlet points and concomitant localized acetate formation (Larsson et al., 1996; Enfors and Häggström, 1999; Enfors et al., 2001; Srivastava and Gupta, 2011). Limitations to the feed control system can also cause unintended exposure to elevated sugar concentrations (Larsson et al., 1996). Finally, if cells grow slower than expected, the feed profile can exceed the threshold of the specific sugar uptake rate that induces overflow metabolism. Consequently, a slight mismatch between growth and feed rate can lead to a negative spiral of growth inhibition and acetate accumulation that quickly result in a lost fermentation batch (Chrapkova et al., 2023). This phenomenon has been observed multiple times with different HMO producing *E. coli* strains at the dsm-firmenich manufacturing site in Esbjerg. It is therefore desirable to have a production strain that is resistant to sugar exposure as it leads to more robust processes. Various genetic engineering strategies have been explored to achieve this. However, while mixed-acid metabolism under anaerobic conditions is well-understood (Clark, 1989; Förster and Gescher, 2014), the phenomenon of overflow metabolism remains more enigmatic despite decades of research. This has increased the difficulty in devising a successful metabolic engineering strategy. Several hypotheses have been proposed to explain the occurrence of acetate overflow metabolism despite it being less energy-efficient compared to respiration. This includes more cost-efficient proteome allocation in fermentation compared to respiration during faster growth rates (Basan et al., 2015), limited capacity of respiration resulting from space limitation in the plasma membrane where the electron transport chain is located (Andersen and von Meyenburg, 1980; Zhuang, Vemuri, and Mahadevan, 2011) and saturation of the tricarboxylic acid cycle (TCA cycle) and/or electron transport chain with sugar influx that surpasses the cells’ biosynthetic demand (De Maeseneire et al., 2006; Vemuri et al., 2006). Several studies have reported on engineered *E. coli* strains with a reduced (Farmer and Liao, 1997; Lin et al., 2006; De Mey, Lequeux, et al., 2007; Lara et al., 2008; Parimi et al., 2017) or abolished (Fuentes et al., 2013) acetate overflow during unrestricted growth. However, unrestricted growth produces a very different physiology compared to carbon limited growth. In addition, industrial strains are often designed such that the expression of the production machinery is coupled to changes in the extracellular environment or growth (reviewed in (Zhu et al., 2012)). A typical example is the use of promoters that are catabolically repressed during non-limited growth but fully expressed and only fully burdening the cell machinery in the carbon-limited production phase (Srivastava and Gupta, 2011; Maury et al., 2018; Pedersen and Papadakis, 2019). Since cells in this phase are in a very different physiological state, with a different metabolic burden, transitioning from carbon limitation to carbon excess can lead to a very different physiological reaction than what can be predicted from data acquired from cultures acclimatized to unrestricted growth. It is therefore useful to include such experiments when evaluating a strain’s robustness.

**FIGURE 1:**
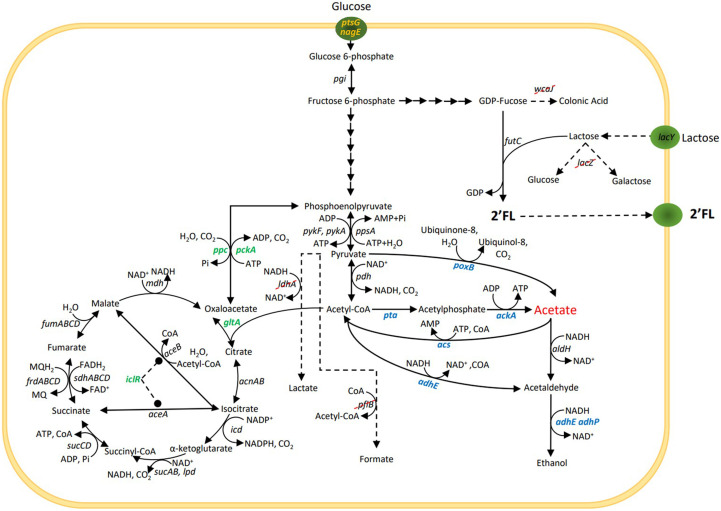
Simplified central carbon metabolism and 2’-*O*-fucosyllactose (2’FL) production pathway in *Escherichia coli*. For the 2’FL production, lactose is used as a sugar acceptor and GDP-fucose (sugar nucleotide) as a sugar donor. Specific glycosyltransferases, e.g., *futC* are then used to transfer the fucose moity from the donor to the acceptor to assemble the HMO. Gene *lacZ* is removed to prevent degradation of lactose and *wcaJ* is removed to increase the GDP-fucose pool. Genes *ldhA* and *pflB* are removed to block mixed-acid fermentation pathways. The three strategies used to reduce acetate production under aerobic conditions and selected genes for these strategies are marked in blue, green and yellow.

This study evaluated how three different metabolic engineering strategies previously shown to reduce acetate accumulation in *E. coli* affected the acetate formation, biomass growth and 2’FL production during either unrestricted growth on glucose or in a glucose-limited process exposed to glucose shock. The three strategies are described below and in Figure 1:

Strategy 1 - Alterations of direct pathways to and from acetate (Dittrich et al., 2008; Phue et al., 2010; Tao, Cheng, and Kopatsis, 2012). This included the deletion of *poxB*—encoding pyruvate oxidase that catalyses the direct conversion of pyruvate to acetate, deletion of *pta*—encoding phosphate acetyltransferase, the first enzyme in the acetyl-CoA to acetate pathway, deletions of *adhE* and *adhP*, encoding two alcohol dehydrogenases involved in ethanol formation from acetyl-CoA and the concomitant oxidation of 2 NADH molecules. While not directly involved in acetate formation, NAD^+^ is needed for acetyl-CoA formation from pyruvate, which could potentially become a limiting factor under conditions when NADH oxidation via oxidative phosphorylation is a bottleneck, such as during overflow metabolism. Reduction of alternative ways to oxidize NADH could thus reduce the acetyl-CoA formation rate and thereby acetate formation. Finally, overexpression of *acs* encoding acetyl-CoA synthetase, a high affinity ATP driven acetate scavenging enzyme, that converts acetate into acetyl-CoA (Lin et al., 2006).

Strategy 2 - Mutations that channel the metabolic flux away from the acetate precursors pyruvate and acetyl-CoA and into the TCA cycle (De Maeseneire et al., 2006; De Mey, Lequeux, et al., 2007; Zhu et al., 2019). This included overexpression of *gltA* encoding citrate synthase, which constitutes the main entry point into the TCA cycle, converting acetyl-CoA and oxaloacetate into citric acid, overexpression of *ppc* that encodes phosphoenolpyruvate carboxylase that is the second entry point into the TCA cycle converting PEP and CO_2_ into oxaloacetate and which also reduces the availability of PEP which is driving the glucose uptake via the PTS system, deletion of *pckA* encoding phosphoenolpyruvate carboxykinase, catalyzing the reverse reaction from oxaloacetate into PEP, deletion of *iclR*, a negative regulator for the glyoxylate bypass. Deletion of *iclR* has been shown to increase the flux through the glyoxylate shunt and increase the overall TCA capacity (Waegeman et al., 2011).

Strategy 3 - Reducing the maximum glucose uptake rate below the threshold that triggers overflow metabolism (Chen et al., 1997; Cho et al., 2005; De Anda et al., 2006). This was attempted by the deletion of the enzyme IIBC components in the PTS system for glucose (encoded by *ptsG*) and N-acetylglucosamine (encoded by *nagE*), which is also able to transport glucose and contributes to growth in Δ*ptsG* genotypes (Fuentes et al., 2013).

These genetic modifications and their combinations were introduced into an industrial 2’FL producing strain (C1) with a mixed-acid negative phenotype that prevent anaerobic metabolism due to an imposed redox imbalance (Δ*pflB*, Δ*ldhA* and Δ*focA* genotype). Together these knockouts prevent the *E. coli* cells from passing the pyruvate to acetyl-CoA node without concomitant NAD^+^ to NADH reduction, and from oxidizing any produced NADH by converting pyruvate into lactate as terminal electron acceptor. Therefore, without oxygen available as electron acceptor, these cells quickly run low on NAD^+^ which halts the metabolism (Clark et al., 1988; Mat-Jan and Clark, 1989; Lara et al., 2006; Wu et al., 2007; Singh, Lynch, and Gill, 2009). After an initial screening in microplates, selected strains were tested in bioreactors in a high-cell density, glucose-limited fed-batch process and challenged by a large bolus addition of glucose to trigger *E. coli*’s overflow metabolism.

## 2 Results

### 2.1 Initial strain characterization in microplate experiments

A microplate assay was used as an initial high-throughput screening tool to narrow down the number of candidate strains before further characterization in bioreactors. The assay was designed to evaluate the impact of various modifications on both acetate formation and growth during non-limited growth and 2’FL production during carbon-limited growth. To properly evaluate the impact on 2’FL formation, carbon-limited growth was needed. Therefore, each strain was tested under two conditions: i) with excess glucose, where the acetate yield from overflow metabolism and their growth rates (µmax) could be estimated, and ii) with a slow and controlled release of glucose and fructose from sucrose using the enzyme sucrose hydrolase to ensure a carbon-limited environment, which is necessary to properly evaluate 2’FL yield.

Among the single modifications tested (see Table 1), only deletion of *poxB* (strategy 1) and overexpression of *gltA* and *ppc* (strategy 2) significantly reduced the acetate yield in the assay. Deletion of the glucose transporters *ptsG* and *nagE* (strategy 3) affected acetate levels, but the Δ*ptsG* modification severely affected the growth rate, which made it difficult to properly quantify the glucose consumption and calculate yields in the microplate assay. Surprisingly, increasing the expression level of *acs* did not have an impact, even though such effects has previously been reported (Lin et al., 2006; Peebo et al., 2014).

**TABLE 1:** Performance of strains engineered to reduce production of acetate in batch (for growth and acetate yield) and enzymatic fed-batch (for 2’FL titers) conditions, performed in microplate experiments. The results are presented in blocks, following the three different strain design strategies: 1) Alterations to pathways to and from acetate, 2) Increasing carbon flux towards TCA cycle and 3) Reducing glucose uptake rate. The values are presented as the average of three individual cultures relative to the C1 control strain. Δ–corresponds to deletion and OE–corresponds to overexpression. ± Standard deviation. *Results with statistical significance (*p* < 0.05).

Strain ID	Relevant genotype	Relative values (%)		
		µmax (h^-1^)	Y_acetate/glucose_ (g/g)	2’FL (g/L)
C1	Industrial 2’FL producer (Control)	100 ± 5	100 ± 6	100 ± 5
Alterations to pathways to and from acetate				
AP1	C1 *Δpta*	76 ± 3 *	112 ± 3 *	106 ± 6
AP2	C1 *ΔpoxB*	96 ± 1	77 ± 2 *	96 ± 1
AP3	C1 *Δpta ΔpoxB*	75 ± 2 *	20 ± 1 *	102 ± 2
AP4	C1 *ΔadhE*	100 ± 2	99 ± 6	101 ± 7
AP5	C1 *Δpta ΔpoxB ΔadhE*	72 ± 2 *	18 ± 2 *	108 ± 6
AP6	C1 *Δpta ΔpoxB ΔadhE ΔadhP*	82 ± 3 *	17 ± 2 *	118 ± 8
AP7	C1 ^*OE*^ *adhE*	100 ± 2	104 ± 2	96 ± 4
AP8	C1 ^*OE*^ *adhP*	97 ± 1	99 ± 4	98 ± 8
AP9	C1 ^*OE*^ *acs*	101 ± 5	101 ± 4	98 ± 6
Increased carbon flux towards TCA cycle				
TCA1	C1 *ΔgltA*	2 ± 3 *	14 ± 3 *	12 ± 3 *
TCA2	C1 ^*OE*^ *gltA*	92 ± 3	87 ± 4 *	95 ± 2
TCA3	C1 ^*OE*^ *ppc*	85 ± 2 *	75 ± 2 *	94 ± 1
TCA4	C1 ^*OE*^ *gltA* ^*OE*^ *ppc*	82 ± 1 *	51 ± 1 *	93 ± 3
TCA5	C1 *ΔiclR*	108 ± 1	95 ± 2	129 ± 4 *
TCA6	C1 *ΔpckA*	101 ± 3	101 ± 5	94 ± 4
TCA7	C1 *ΔpckA ΔiclR*	106 ± 1	92 ± 3	124 ± 2 *
TCA8	C1 ^*OE*^ *gltA ΔiclR*	95 ± 3	27 ± 2 *	123 ± 5 *
APTCA1	C1 *Δpta ΔiclR*	80 ± 3 *	108 ± 1	129 ± 6 *
APTCA2	C1 *Δpta ΔpoxB ΔiclR*	83 ± 2 *	17 ± 2 *	130 ± 6 *
Reduced glucose uptake rate				
GU1	C1 *ΔptsG*	13 ± 6 *	15 ± 1 *	86 ± 6 *
GU2	C1 *ΔptsG ΔnagE*	5 ± 1 *	13 ± 3 *	85 ± 13

Estimating the acetate yields of mutants with reduced glucose uptake rates was challenging due to the slower growth and lower acetate numbers at the harvest point. Deletion of the *ptsG* gene (GU1) resulted in an 87% decrease in growth rate and acetate levels that were difficult to quantify after the 24-h batch experiment. The small decrease in 2’FL titer (−14%) was more easily determined as the experiment was performed in a 48-h fed-batch assay. Similarly, the subsequent deletion of the *nagE* gene (GU2) resulted in a 95% reduction in growth rate. The effect on acetate yield and 2’FL titer was comparable to the GU1 mutant. Considering the impact on growth, it was not possible to draw any firm conclusion about GU1 and GU2 in the microplate experiment, other than the fact that the growth rate had been reduced.

Several combined modifications produced synergistic effects. Deletion of *poxB* produced a modest reduction of acetate by 23%, whereas *pta* deletion caused a slight increase. However, in combination, they reduced the acetate yield by 80%. Moreover, increasing the expression levels of the two entry points into the TCA, *gltA* and *ppc*, caused only a small reduction in acetate formation (−13% and −25%, respectively), but the combined effect of the mutations was more than additive (−49%). Combining *gltA* overexpression with Δ*iclR*, which by itself had no impact on the acetate yield, had a significant impact (−73%). Interestingly, all strains with *iclR* deletion had a 20%–30% increase in 2’FL production.

### 2.2 Characterization of selected mutants in a bioreactor system

**FIGURE 2:**
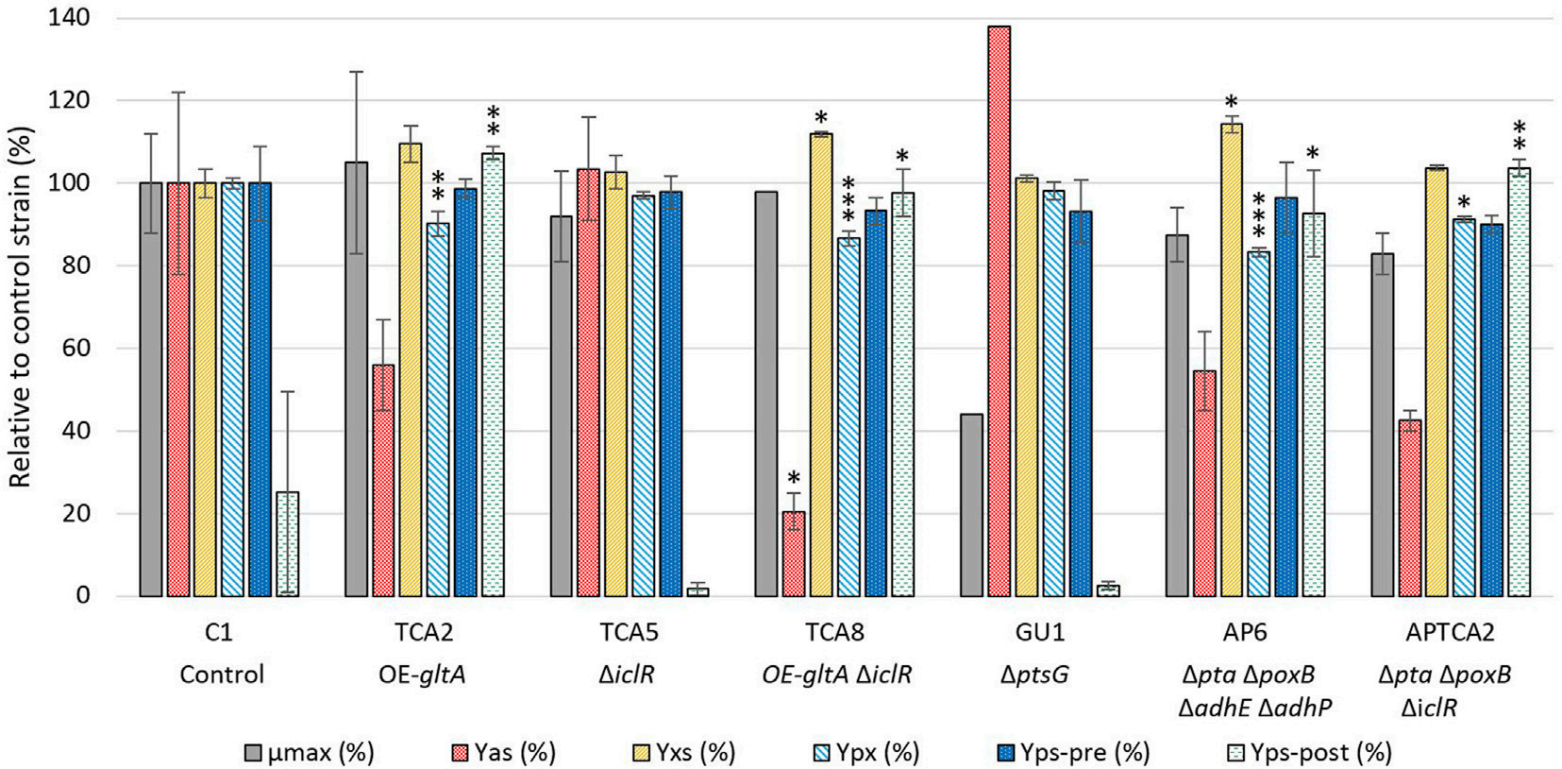
Comparison of strain performance in bioreactors. Samples for maximum growth rate (µmax) and acetate yield (Yas) determination were taken at the end of the batch phase, while samples for biomass yield (Yxs) and biomass specific product yield (Ypx) were taken before the glucose pulse addition, approximately 43–48 h after the start of the feed phase. Separate product yields were calculated before and after the glucose pulse addition (Yps-pre and Yps-post) from the linear slope of the 2’FL accumulation/glucose consumption. The values are relative to the control strain (C1), except for the Yps-post, which is relative to the C1 strain prior to the glucose pulse. All values are given as percentages. The fermentation with the control strain and glucose pulse addition was performed in biological triplicate (n = 3, error bars = standard deviation), whereas the control strain without pulse addition and the mutants were performed in biological duplicate (n = 2, error bars = range). Note that for some strains there were only one datapoint available for µmax (TCA8, GU1) and Yas (GU1). A statistical analysis was done comparing results from the engineered strains to C1 using one-way ANOVA with Dunnett post-test. Significance levels are indicated with * above the columns, where * = *p* ≤ 0.05, ** = *p* ≤ 0.01, *** = *p* ≤ 0.001.

The strains that showed the lowest acetate formation without having a negative effect on 2’FL production were selected for further study in a 250 mL bioreactor system where dissolved oxygen (DO), pH and glucose feed rate could be controlled. A total of 6 mutants were tested and compared to the C1 control strain in at least two biological replicates. These mutants included TCA2, GU1, TCA8, TCA5, APTCA2 and AP6, see Figure 2. Despite the Δ*ptsG* strain having a moderately reduced 2’FL titer, it was included in the fermentation experiments where the growth conditions could be better controlled. TCA2 and TCA8 were selected to evaluate the beneficial effect of the *iclR* deletion in combination with *gltA* overexpression on 2’FL titer and lower acetate yield compared to other *gltA* and *ppc* combinations (Table 1). The maximum growth rate (µmax) and acetate yield (Yas) were determined in the batch phase, and the product yield (Yps), biomass yield (Yxs) and biomass specific product yield (Ypx) were determined in the fed-batch phase (Figure 2). The maximum growth rates (µmax) of the mutant strains were estimated from the development of the optical density in the batch phase until glucose depletion, which occurred at an OD_600_ = 30 ± 2.

The growth of all mutant strains were comparable to the control C1 strain, except the GU1 strain, which was significantly slower (Figure 2). The growth rate of GU1 was higher in the fermenter than in the microplate assay, but the relative impact compared to the control was greater than what had been reported previously (Fuentes et al., 2013).

Acetate measurements showed that the control strain produced up to 1.5 g/L of acetate at the end of the batch phase (OD_600_ = 30). The highest acetate level overall was observed with the *ptsG* deleted GU1 strain, which accumulated up to 1.8 g/L at the end of the batch phase (OD_600_ = 28). The TCA8 strain carrying the *gltA* overexpression and *iclR* deletion had the lowest accumulation of acetate (0.19 g/L). Strains with multiple deletions such as AP6 and APTCA2, as well as strain TCA2 reached up to 0.5 g/L of acetate. Interestingly, organic acid quantification revealed glutamate accumulation up to 3 g/L but with a very high batch-to-batch variation (Supplementary Table S1).

**FIGURE 3:**
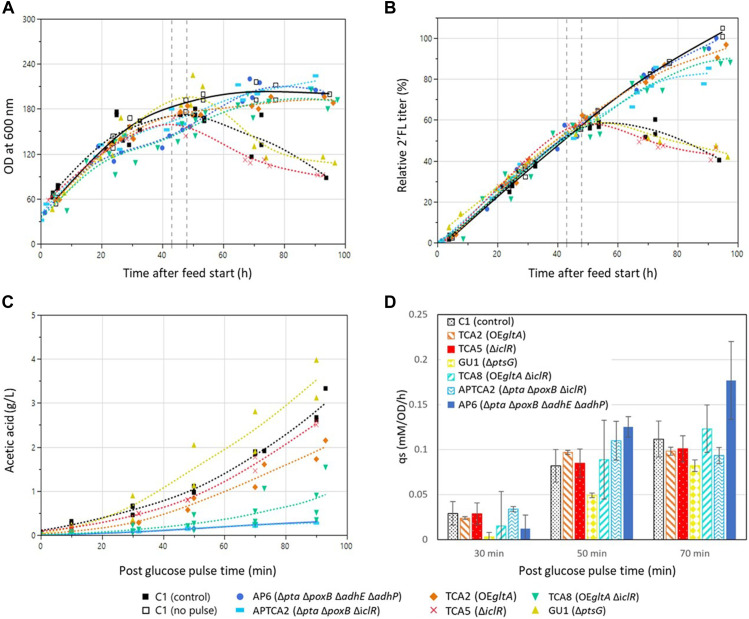
Fed-batch fermentation development over time before and after the addition of a glucose pulse. Genotypes and marker shapes for the control and mutant strains are shown below the figure. Trendlines for fermentations with the control strain with (closed squares, dashed line) and a reference without a glucose pulse (open squares, solid line) are shown in black. The time window 43–48 h into the feed phase in which the glucose pulses were added is marked with dashed grey vertical lines. **(A)** Cell density development in the fed-batch fermentations of control and mutant strains. **(B)** 2’FL concentration in the fed-batch fermentations of control and mutant strains. Values are shown relative to the highest value in the dataset of the pulsed fermentations. **(C)** acetic acid development in the fed-batch experiments after glucose pulse addition. Samples were collected and acetic acid was quantified before the pulse addition (t = −5 min), at the time of addition (t = 0) and at intervals until 90 min. Mutant strain experiments were performed in duplicate (n = 2) whereas the control strain experiment was performed in triplicate (n = 3). **(D)** Biomass specific glucose consumption rate (q_s_) after the addition of the large glucose pulse. The q_s_ was calculated as incremental differences between sample points. Experiments with mutants were performed in duplicate except the control strain which was in triplicate. Error bars indicate the standard deviation for the control strain and the range of the duplicates for the mutants.

In general, the 2’FL titer development prior to the glucose pulse were found to be similar for all strains (Figure 3B) and a statistical analysis on the 2’FL yield on glucose (Yps-pre (%) in Figure 2) did not reveal any significant difference in any mutant strain compared to the control. However, the calculated biomass yield on glucose (Yxs) was higher for some of the mutant strains. Given the variation in biomass yield, the biomass-specific 2’FL yield (Ypx) was also compared between the strains. TCA2, TCA8, AP6 and APTCA2 all had significantly lower Ypx than C1. The largest reductions were observed for TCA8 (reduced by 12%–15%) and for AP6 (reduced by 16%–18%). The reduction was predominantly caused by their increased biomass levels. In combination with slightly lower meased Yps it resulted in significantly reduced Ypx.

### 2.3 Stress testing of selected mutants

To test the strains’ ability to withstand a sudden glucose shock during glucose-limited growth, a single glucose bolus addition corresponding to a final concentration of 10 g/L (55 mM) was added 43–48 h into the fed-batch phase. The size of the glucose addition was chosen to pose a greater challenge than previous studies where *E. coli* had been exposed to glucose pulses ranging from 0.08 to 3.7 g/L, which had triggered overflow metabolism but not destabilized the growth of the cultures (Enfors and Haggstrom, 1999; Sunya et al., 2012). In addition to a larger pulse, a higher cell density was also used (50 g/L dry weight compared to 4–10 g/L dry weight). Samples were withdrawn for organic acid and glucose measurements before and after the pulse (at 0, 10, 30, 50, 70, 90 and 120 min). Air sparging and stirring were set to the maximum before the glucose pulse additions to prevent limiting DO levels for extended periods. DO levels did still drop to 0% in some of the vessels, but only for a brief amount of time (few minutes), which meant it could not contribute significantly to the overall acetate levels based on the acetate production rates (Supplementary Figure S2).

In all experiments all glucose was consumed within 70 min of the pulse and the highest biomass specific glucose consumption rates (qs) were measured between 30 and 50 min after the pulse. (Supplementary Figure S4). The *ptsG* deleted GU1 had a decreased qs, whereas AP6 had the highest (Figure 3D).

Acetate accumulation was observed in all cultures following the glucose pulse (Figure 3C). The two Δ*pta* Δ*poxB* strains AP6 and APTCA2 showed the lowest accumulation, reaching 0.3 g/L after 90 min when all the glucose had been consumed, this corresponded to only 10% of the 3 g/L the control accumulated (Supplementary Figure S4). The *gltA* overexpression strains TCA2 and TCA8 also produced less acetate than the control strain, reaching 1–2 g/L. On the other hand, TCA5 and GU1 accumulated close to 3 g/L of acetate after 90 min, which was the same as the control strain C1. Glutamate only accumulated when using one strain—the *gltA* overexpressing strain TCA2, which reached 1–1.25 g/L when measured 90 min after the glucose pulse (Supplementary Figure S3). No glutamate production was observed in the strain combining deletion of *iclR* with *gltA* overexpression (TCA8) indicating that deletion of *iclR* can counteract glutamate accumulation caused by *gltA* overexpression.

**FIGURE 4:**
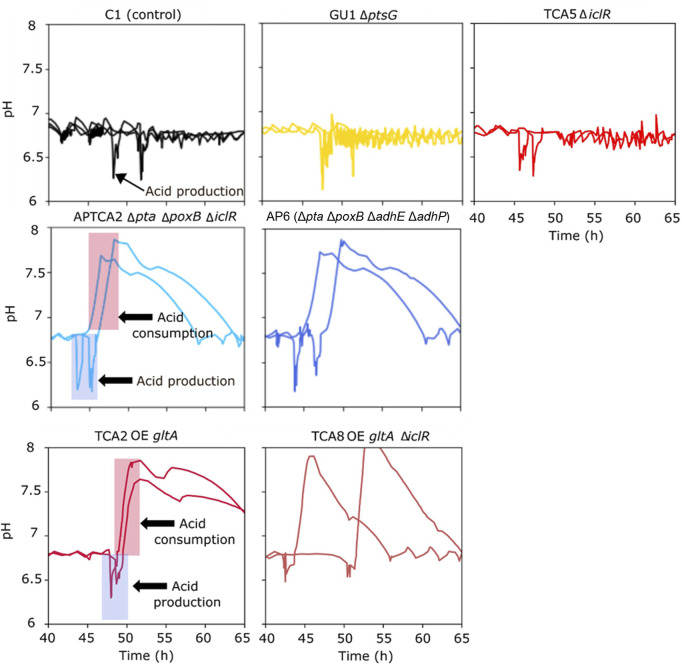
Online measurements from fed-batch fermentations showing pH values before and after a glucose bolus addition. Each graph shows the results from duplicate fermentations. The period with rising pH indicates the window where acids generated from the pulse were re-consumed (marked with arrows in the two examples).

Following the addition of the glucose pulse, all strains produced organic acids (acetate), resulting in an intermittent pH drop (Figure 4). Many of the strains were able to re-assimilate the produced acids, but the control strain and mutant strains that accumulated high levels of acetate were later unable to restore growth or production of 2’FL (Figure 3). This was also evident from the pH profiles as pH in this experiment was only controlled by base titration. The acidifying effect of any organic acids produced were thus neutralized by base addition, but in the cases the acids were re-consumed it could not be compensated by acid titration. This could be seen as a substantial raise in pH (in some cases to almost pH 8) in the cultivations that survived the glucose pulse (Figure 4). Based on these pH profiles, it was evident that TCA2, TCA8, APTCA2 and AP6 re-consumed the produced acids. On the other hand, no pH peak was seen in the fermentations of TCA5 and GU1 or the control strains (Figure 4), indicating that accumulated acids were not being re-consumed. Moreover, the biomass development and 2’FL production showed that TCA5 and GU1 stopped growing and producing 2’FL after the addition of the glucose pulse (Figures 2, 3B). The control strain C1 was also severely affected, whereas strains TCA2, TCA8, APTCA2 and AP6 were all able to survive, continue to grow and produce 2’FL (Figures 2, 3A, B) with high yield.

## 3 Discussion

The tendency of *E. coli* to revert to overflow metabolism has long been a challenge for biotechnological applications and it has therefore remained an attractive target for metabolic engineering (reviewed by Eiteman and Altman, 2006; De Mey; De Maeseneire et al., 2006) Many strategies have been proposed and implemented, but with the focus on the effect during prolonged batch growth. Here we compare the effect of three distinct approaches and some combinations thereof in a commercial production strain: 1) alterations to acetate pathways, 2) increased flux towards the TCA cycle, and 3) reduced glucose uptake rate. The strain used for the study redirects a large proportion of the glucose carbon source towards 2’FL production, which exhorts an additional metabolic load adding to the overall stress level, something not typically taken into account in the published studies. In addition to batch growth, we also show the effect of the genetic modifications when transitioning from carbon limited to unrestricted growth, something hitherto unreported. This is especially relevant in industrial fed-batch and continuous fermentations which are utilizing carbon limitation but are exposed to local sugar gradients at the feed inlet zones or could be exposed to sudden increases in sugar concentration due to constraints in the manufacturing setup (Larsson et al., 1996).

To ensure that the genetic modifications did not compromise the general performance of the strains, we first screened them in a microtiter plate assay. The only strains that showed a significant decrease in 2’FL production were the ones with the *ptsG* deletion (Table 1). The *iclR* deletion strains, on the other hand, exhibited an enhanced 2’FL yield. However, these results were not confirmed in subsequent fed-batch fermentations with selected strains under glucose-limited conditions. Neither the *ptsG* nor the *iclR* deletion had any measurable effect on growth or 2’FL yield in the bioreactors under controlled fermentation conditions. We attribute the discrepancy between the microtiter plate and the bioreactor experiments to two factors: the inoculation ratio and the pH control. The strains carrying the *ptsG* deletion had a lower initial biomass in the microtiter plate assay due to their reduced growth rate in the previous step and the lack of OD_600_ normalization in the inoculation step. This could have influenced the outcome of the assay. The *iclR* deletion strains on the other hand might have been less impacted by the lack of pH control in the microtiter plate assay compared to the other strains. While the strains mixed-acid negative phenotype is very effective during anaerobic conditions (Clark et al., 1988; Mat-Jan and Clark, 1989; Singh, Lynch, and Gill, 2009), we have observed that acid formation occur under microaerobic conditions, probably due to restoration of some of the redox imbalance via respiration while still maintaining a fermentative metabolism. The microtiter experiments turn microaerobic over time and due to the lack of a pH controller system the broth therefore acidify over time. This eventually leads to growth inhibition and cessation of product formation. The *iclR* deletion mutants all showed reduced glutamate accumulation during the glucose stress test (Supplementary Figure S3). If the deletion also affected acid formation in the microplate assay it could have delayed the acidification of the medium and thereby prolonged the productive phase and increased the 2’FL titer.

In contrast to the fed-batch test, the batch tests produced marked differences in both growth rate and acetate yield for some of the genetic modifications. This was seen in both the microtiter assay and the bioreactors. There were two combinations with especially noteworthy synergistic effects. The first was the combined disruption of *pta* and *poxB*. In the microtiter experiment, the disruption of the acetate pathway from acetyl-CoA by *pta* deletion reduced the growth rate and led to an increase in acetate formation (+12%), while the disruption of the acetate pathway from pyruvate via *poxB* deletion only resulted in a minor reduction in acetate accumulation (−23%). However, together they had a marked synergistic effect and produced a five-fold reduction (−80%). The second was the combination of *gltA* overexpression and *iclR* deletion. Overexpression of the *gltA* gene has previously been shown to effectively eliminate acetate production by intensifying the flux through the TCA cycle (De Maeseneire et al., 2006) and to increase anaplerotic reactions (Im et al., 2020). Although *gltA* overexpression did not reduce acetate to such an extent in our experiments (−13% and −44% in microplate assay and bioreactor, respectively), its overexpression together with the increased uptake capacity in the TCA cycle with the *iclR* deletion (Waegeman et al., 2011), which by itself had a negligible effect, produced the overall biggest reduction in acetate accumulation of any strain in the bioreactor batch phase (−80%). In contrast, the combination of the *iclR* deletion with the *pta* and *poxB* deletions did not produce a significant difference. The effect of Δ*iclR* indicated that the capacity of the TCA cycle had become a bottleneck when the entry point (*gltA*) was overexpressed. By creating a shortcut in the glyoxylate shunt, this limitation was relieved and the overall capacity from acetyl-CoA to isocitrate increased and more flux was channelled away from acetate formation (Figure 1). The underlying reason could either be a direct rate limitation in one of the enzymatic steps in the TCA cycle, or a limitation in respiration and its ability to oxidize NADH/FADH_2_, a demand that is lessened if the glyoxylate shunt is increased as fewer NADH equivalents are produced (Waegeman et al., 2011).

The glucose pulse addition experiment constituted a stress test with a direct transition from glucose limited growth with its characteristic physiology to unrestricted growth - without any adaptation time. Although none of the modifications eliminated overflow metabolism from the freely available glucose, strains with specific genetic modifications accumulated much less acetate and showed an increased robustness. The reduced acetate formation rate in these strains seems to have prevented the accumulation from reaching a critical level, which would otherwise cause a viscous cycle of ever-increasing acetate production and growth inhibition (Millard et al., 2021). In contrast, cultures lacking these modifications kept accumulating acetate until they ceased to grow and produce (Figure 2). Furthermore, all strains that survived the glucose pulse showed an increase in pH, indicating the reassimilation of the accumulated acetate (Figure 3). These findings reflect a previous study that demonstrated that engineered *E. coli* strains with a reduced acetate phenotype can reduce fermentation failures caused by acetate accumulation (Chong et al., 2013).

Interestingly, in the bioreactor batch experiment the TCA8 strain with *gltA* overexpression and *iclR* deletion accumulated significantly less acetate than the Δ*pta* Δ*poxB* strains AP6 and APTCA2. This relation completely shifted in the pulse experiment where the AP6 and APTCA2 strains accumulated much less acetate than TCA8. This discrepancy shows the importance of considering the prior physiological state when trying to predict a reaction to a particular change. In this case, it meant that learnings of how particular genetic modifications affected acetate formation during unrestricted growth in the batch phase could not be directly applied to the response to a glucose pulse in a fed-batch phase - even if the pulse led to unrestricted growth. When cells are exposed to such a sudden change, they do not have time to adapt, and therefore their previous physiological state will influence how they react (Vasilakou et al., 2020). The difference in physiology in carbon-limited versus unrestricted growth carried over and made the cultures react differently than what would be expected from the batch experiments alone.

Inactivation of the glucose component of PTS by deletion of *ptsG* to reduce the phosphoenolpyruvate (PEP)-dependent glucose transport rate was expected to be a very efficient way to reduce acetate overflow metabolism based on previous reports (Sigüenza et al., 1999; De Mey, De Maeseneire, et al., 2007; Lara et al., 2008; Fuentes et al., 2013). *E. coli* is known to possess several alternative transporters for glucose uptake (Boos and Howard, 1998; Hua et al., 2004), including alternative PTS system for mannose (encoded by *manXYZ*) and GlcNAc (encoded by *nagE*) that can take up glucose at a reduced rate (Fuentes et al., 2013; Crigler et al., 2018), as well as non-PTS systems such as galactose permease (encoded by *galP*), galactose ABC transporter (encoded by *mglBAC*) or maltose ABC transporter (encoded by *malEFG*) (Boos and Howard, 1998; Hua et al., 2004; Fuentes et al., 2013; Crigler et al., 2018). Since these alternative transporters operate at a lower rate, they should avoid overloading the TCA cycle or respiratory capacity and thereby prevent, or at least reduce, the accumulation of acetate from overflow metabolism. Surprisingly, the deletion of *ptsG* did not reduce the acetate yield in the microplate experiment or during batch growth in the bioreactor, and it did not reduce acetate and prevent culture failure during the stress test with the glucose pulse. A lower glucose consumption rate was observed in the mutant, indicating a reduced glycolytic flux and a successful knockout. In theory, this should have resulted in a low acetate overflow phenotype since the TCA cycle and the respiratory capacity had not been changed. This outcome, which is both counterintuitive and different compared to previous studies, is not straightforward to explain. A full genome sequencing could reveal if there were any unintended changes that was introduced during the genetic engineering work, but this was outside the scope of the work.

An alternative strategy to further reduce the flow to pyruvate (and, therefore, acetate) could be to completely knockout the PTS system and overexpress an alternative glucose transporter such as galactose permease (De Anda et al., 2006) or the maltose ABC transporter (Carreón-Rodríguez et al., 2023). In addition to slowing down the glucose uptake rate, non-PTS sugar transporters avoid the conversion of PEP to pyruvate during glucose transport (De Anda et al., 2006), which is a precursor for acetate. Such an approach was able to restore growth while maintaining reduced acetate secretion when *galP* was overexpressed in a *ptsG* mutant (De Anda et al., 2006). Though, it remains to be seen if such a strategy would be successful with an industrial 2’FL producing strain.

A recent study showed that overflow metabolism confers a competitive advantage over complete respiration (Rabbers et al., 2022) suggesting an evolutionary role for acetate cycling. Complete elimination of acetate formation may therefore inherently result in trade-offs, such as slower growth, the accumulation of other by-products, or sensitivity to other process conditions that only a very extensive rewiring of the *E. coli* metabolism might be able to solve.

## 4 Conclusion

The findings herein show that blocking the major acetate pathways and increasing the flux towards the TCA cycle can contribute to a reduced acetate phenotype for both regular batch cultures and for glucose limited cultures transitioning to non-limited growth. The genetic modifications were shown to enhance cell robustness, enabling an industrial strain to better tolerate processes exposed to dramatic changes in glucose concentration. The best performers in terms of 2’FL yield after a glucose shock were the strains that overexpressed *gltA*, and the ones carrying the combined *pta*, *poxB* deletions. The *iclR* deletion, while not effective on its own, reduced acetate formation when combined with *gltA* overexpression, and it counteracted the tendency of glutamate accumulation caused by the *gltA* overexpression. Future work for the development of glucose gradient resistant production strains could explore these two strategies, as well as the combination of them (Δ*pta* Δ*poxB* with OE*gltA* and Δ*iclR*). It is also recommended to follow up with a study of the impact of repeated stress, such as in a pulse-fed scale-down reactor (Vasilakou et al., 2020), as this could have a different effect on the physiology and possibly differentiate the performance of the strains. Finally, as an attempt to understand why the *ptsG* deletion was not effective in reducing acetate formation in this work, a comparison of the metabolic and transcriptomic expression profiles of a *ΔptsG* strain with a low acetate phenotype as reported in previous studies would be interesting.

## 5 Methods and materials

### 5.1 Bacterial strains

The background strain used for the construction of reduced-acetate phenotype mutants was derived from *E*. *coli* K-12 DH1 with the genotype: Fˉ, ʎ-, gyrA96, recA1, relA1, endA1, thi-1, hsdR17, supE44. This strain was further modified to generate the 2’FL producing C1 strain. The modifications included deletion of *lacZ*, *lacA*, *wcaJ*, *mdoH*, overexpression of one additional chromosomally integrated copy of the colonic acid operon *gmd-fcl-gmm-wcaI-manC-manB* under the control of the synthetic expression element PglpF (Pedersen and Papadakis, 2019) and overexpression of two chromosomally integrated copies of alpha-1,2-fucosyltransferase (encoded by *futC*) from *Helicobacter pylori* 26,695 (homologous to NCBI Accession nr. WP_080473865.1), also under the control of PglpF. Furthermore, the strain was modified with deletion of *pflB*, *ldhA* and *focA* genes to block mixed-acid fermentation pathways under anaerobic conditions. All strains used in the present study are listed in Table 1. The gene deletions were carried out in two steps. Firstly, the gene of interest was replaced by the genetic cassette CP6-galK. The *galK* gene, controlled by the CP6 promoter, was amplified from the pT7-CP6-galK plasmid (Richter 2014) using primers O982 and O983 (Supplementary Table S2). A double stranded DNA fragment covering CP6-*galK* with flanking regions homologous to the DNA sequences located upstream and downstream of the gene of interest (required for homologous recombination) was constructed by PCR. The amplified CP6-*galK* DNA, flanked by the homologous DNA sequence upstream and downstream of the gene of interest, was used for double strand recombineering. This was done as described elsewhere (Datsenko and Wanner, 2000) using helper plasmid pACBSR (Herring, Glasner, and Blattner, 2003). In the second recombineering step, CP6-*galK* was removed using single-stranded DNA recombineering (Sawitzke et al., 2013) (Supplementary Table S2) and the helper plasmid pACBSR. Strain selection was performed as described elsewhere (Warming et al., 2005). Gene deletions covered the entire coding sequence of the gene, including translational start codon and stop codon. Gene overexpression’s were achieved by replacing native gene promoters with synthetic expression elements such as PglpF, PglpF_SD7 (Pedersen and Papadakis, 2019) or Pcon3_70UTR. The replacement of native promoter sequences upstream of genes was performed in two steps, as described above. The promoter sequences (PglpF, PglpF_SD7 and Pcon3_70UTR) inserted upstream of the genes are listed in Supplementary Table S2.

### 5.2 Microplate assays

Microplate experiments were used for initial screening of growth, acetate and 2’FL production. Basal minimum medium (BMM) was prepared by autoclaving 7 g/L KH_2_PO_4_, 7 g/L NH_4_H_2_PO_4_, 2.5 g/L KOH, 1 g/L NaOH, 0.5 g/L citric acid, and trace metal solution. The pH was adjusted to pH 7.0 or pH 7.5 with 5N NaOH. The precultures were grown in 96 well plates (Axygen 2 mL PP square well) containing BMM medium (pH 7.0) supplemented with 25 g/L glucose, 1 g/L MgSO_4_ and 4 mg/L thiamine. The plates were sealed with a hydrophobic gas-permeable adhesive seal (Axygen) and incubated at 34°C, 1,000 rpm, overnight. For growth characterization, overnight precultures were transferred into microplates containing 200 μL BMM medium (pH 7.5) with 4 mg/L thiamine, 1 g/L MgSO_4_ and 25 g/L glucose. The plates were sealed and incubated at 30°C, 840 rpm for 24 h in a Varioskan™ (Thermo Fisher). Absorbance was measured every 30 min at 600 nm. For characterization of 2’FL production, the overnight precultures were transferred into microplates containing 750 μL BMM medium (pH 7.5) with 50 g/L lactose monohydrate, 37.5 g/L sucrose, 1 g/L MgSO_4_, 0.1 g/L glucose, 2.5 mL/L of 0.1 g/L sucrose hydrolase (SUH) stock solution (300 U/mg invertase from baker’s yeast, Cat. Nr. I4504 Sigma-Aldrich.) and 4 mg/L thiamine. The microplates were then sealed and incubated at 30°C, 1,000 rpm for 48 h. For characterization of acetate production, overnight precultures were transferred into microplates containing 750 μL BMM medium (pH 7.5) with 25 g/L glucose, 1 g/L MgSO_4_ and 4 mg/L thiamine. The plates were sealed and incubated at 30°C, 1,000 rpm for 24 h. For analysis of acetate and 2’FL, the plates were lysed by boiling at 100°C in a shaking incubator at 800 rpm for 1 h followed by incubation at room temperature for 20 min. The boiled and cooled samples were harvested by centrifugation at 4°C, 4,700 g for 10 min and stored at −20°C until further analysis.

### 5.3 Bioreactor cultivations

Precultures for fermentations were grown in two steps. Firstly, strains from glycerol stocks were inoculated into 10 mL glucose minimal medium (GMM) in 50 mL Falcon tubes. The GMM medium was prepared by autoclaving 10 g/L NH_4_H_2_PO_4_, 5 g/L KH_2_PO_4_, 5 g/L K_2_SO_4_, 2.35 g/L NaOH, 1.65 g/L KOH, 1 g/L citric acid and trace metals and supplementing with sterile 10 g/L glucose, 1 g/L MgSO_4_ and 4 mg/L thiamine. The pH of the medium was adjusted to 7.0 with 5N NaOH. The cultures were incubated overnight in a shake-incubator at 33°C, 200 rpm. Aliquots of the cultures were then transferred into 50 mL of fresh GMM medium in 250 mL baffled shake-flasks to a final OD_600_ of 0.3–0.5. The shake-flasks cultures very subsequently grown at 33°C, 200 rpm to an OD_600_ of 2–3.5. These cultures were then used to inoculate the fermenters at an inoculation ratio of 2%. High cell density, glucose-limited, fed-batch fermentations were carried out in 250 mL DASbox^®^ Mini Bioreactor System (Eppendorf) equipped with Hamilton pH and pO_2_ probes, starting with an initial volume of 100 mL. The fermentation medium composed of batch sterilized 5 g/L NH_4_H_2_PO_4_, 5 g/L KH_2_PO_4_, 5 g/L K_2_SO_4_, 0.65 g/L NaOH, 1.65 g/L KOH, 0.5 g/L citric acid, 200 mg/L antifoam 204 (Sigma), trace metals, and sterilized separately, 25 g/L glucose, lactose, 1.2 g/L MgSO_4_, and 8 mg/L thiamine. The dissolved oxygen (DO) was kept at 23% by an agitation (700–2000 rpm) and airflow (1 VVM (L/L/min) cascade. The pH level was maintained at 6.8 by titration with 14% NH_4_OH. All cultures were started with an initial batch phase at 33°C, but the temperature was then lowered to 30°C 3 h after feed start using a 1-h ramp. The feed was triggered by an increase in pH, which appeared when all the initial glucose had been consumed. The feed solution composed of glucose, lactose MgSO_4_, citric acid, trace mineral solution and antifoam 204 and was fed at a constant rate corresponding to 0.437 g glucose/h. Samples were withdrawn regularly from the fermentation broth for 2’FL, lactose, bio-wet mass (BWM) and OD (600 nm) off-line measurements.

#### 5.3.1 Stress test with glucose pulse

The stress test composed of a glucose pulse that was added to the bioreactors approximately 43–48 h after the feed start by a fast injection of a sterile 50% glucose solution to obtain 10 g/L glucose concentration in the fermenters. Samples for organic acids and glucose were withdrawn using a syringe and the cells were immediately removed by filtration through a sterile syringe filter (0.45µm, 33 mm ø). Samples were taken before the pulse, right after the pulse and further at 10, 30, 50, 70 and 90 min after the pulse.

### 5.4 Analytical methods

#### 5.4.1 2’FL and glucose measurements

Fermentation broth samples for quantification of 2’FL and glucose concentrations were immediately spun down by centrifugation at 13,300 g for 3 min at room temperature. The supernatants were analysed with high-performance liquid chromatography (HPLC) (Dionex Ultimate 3000 RS, Supelco TSKgel Amide-80 HPLC column). The solvent was isocratic with 68% acetonitrile and 32% water.

#### 5.4.2 Acetate quantification from microplate experiments

Acetate concentrations were measured from the supernatant using an acetic acid assay kit (Megazyme), following the protocol of the manufacturer.

#### 5.4.3 Organic acid measurement from bioreactor experiments

Organic acids were quantified by NMR at DSM Science and Innovation, Biodata and Translation, Center for Analytical Innovation (Delft, Netherlands). For the quantification of acetate, pyruvate, citrate, fumarate, succinate and glutamate, 70 µL of supernatants were homogenized with 100 μL internal standard solution (adjusted to pH 7.0 with NaOH in deionized water) containing 2 g/L of difluoro-trimethylsilanylmethyl-phosphonic acid. The samples were dried using a stream of nitrogen and subsequently dissolved in 650 µL deuterium oxide (D_2_O).

1D 1H NMR spectra of the clear solutions were recorded on a Avance III HD spectrometer (Bruker BioSpin AG, Fällanden, Switzerland), operating at a proton frequency of 600 MHz, equipped with a cryo probe, using pulse program ZGPR with a solvent suppression power level corresponding to 5 Hz at a temperature of 293K, excitation pulse of 7.9 μs, an acquisition time of 2.3 s and a relaxation delay of 30 s. The number of scans was set at 8, dummy scans were not used.

### 5.5 Statistical analyses

The significance of the microplate experiment (Table 1) was evaluated using a two-tailed, unpaired *t*-test with a significance level of *p* ≤ 0.05, comparing the performance of the engineered strains to C1.

Statistical analysis of key performance indicators of the bioreactor cultivations pre- and post-glucose pulse was performed with GraphPad Prism 9.1.0. using one-way ANOVA with Dunnett post-test.

## Figures and Tables

**Table:** 

**HMO**	Human milk oligosaccharide
**2’FL**	2’-*O*-fucosyllactose
**TCA cycle**	Tricarboxylic acid cycle
**PTS**	Phosphotransferase System
**µmax**	Maximum specific growth rate (h^−1^)
**Yas**	Acetate yield (g acetate/g glucose)
**Yps**	Product yield (g 2’FL/g glucose)
**Ypx**	Specific product yield (g 2’FL/g biomass)
**Yxs**	Biomass yield (g dry biomass/g glucose)
**qs**	Specific substrate consumption rate (g glucose/OD/h)
**DO**	Dissolved oxygen content in the broth (% of saturation in the medium at atmospheric pressure and 33°C)
**OD**	Optical density measured at 600 nm in 1 cm light path
**PEP**	Phosphoenolpyruvate

## Data Availability

The original contributions presented in the study are included in the article/Supplementary Material, further inquiries can be directed to the corresponding author.
