# Supplementary material for: Minimizing acetate formation from overflow metabolism in Escherichia coli: comparison of genetic engineering strategies to improve robustness toward sugar gradients in large-scale fermentation processes
Source: Front Bioeng Biotechnol. 2024 Feb 14;12:1339054. doi: 10.3389/fbioe.2024.1339054 (PMC10899681; doi:10.3389/fbioe.2024.1339054)
Supplement: Supplementary file 1 [file DataSheet4.docx]

**
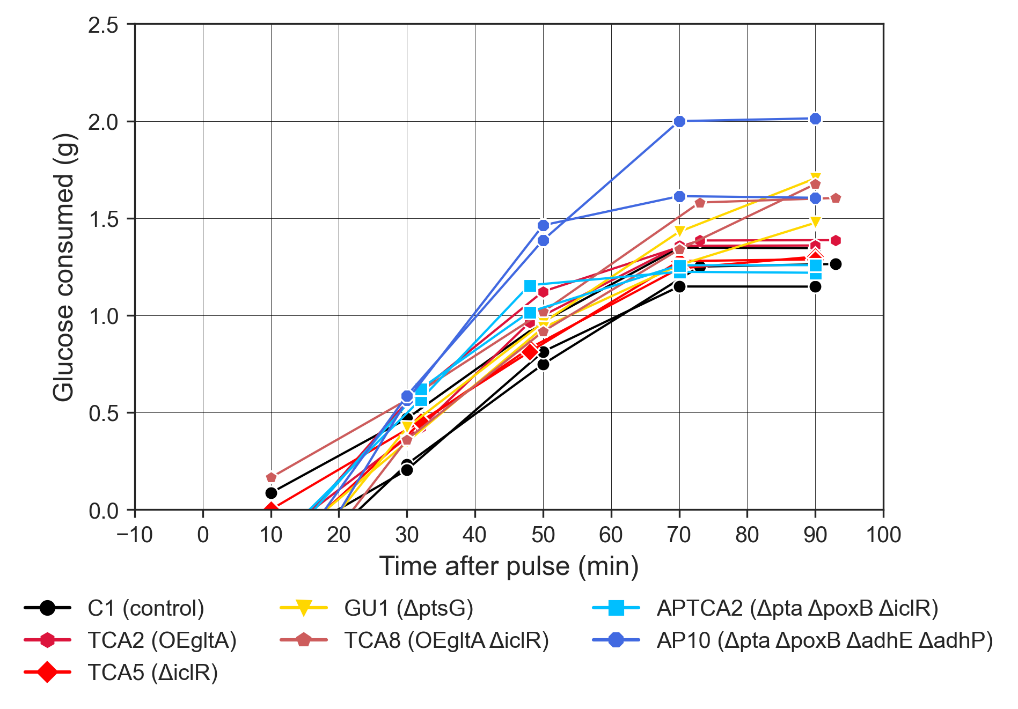
Figure S4.** Accumulated glucose consumption after the addition of a 10 g/L glucose pulse in the bioreactor experiments. The bolus addition of glucose corresponded to approximately 1.5 gram.
