# Supplementary material for: Minimizing acetate formation from overflow metabolism in Escherichia coli: comparison of genetic engineering strategies to improve robustness toward sugar gradients in large-scale fermentation processes
Source: Front Bioeng Biotechnol. 2024 Feb 14;12:1339054. doi: 10.3389/fbioe.2024.1339054 (PMC10899681; doi:10.3389/fbioe.2024.1339054)
Supplement: Supplementary file 2 [file Table1.docx]

Table S1. Overview of the key strain performance indicators in the batch phase of the bioreactor experiments. The range of duplicate experiments are shown.

| **Strain ID** | **Genotype** | **µ_max_**  **(% of C1)** | **Y_acetate/glucose_**  **(% of C1)** | **Y_glutamate/glucose_**  **(% of C1)** | **qs (g glc/h/OD)**  **(% of C1)** |
| --- | --- | --- | --- | --- | --- |
| C1 | Control | 88-112 | 78-122 | 100 | 77-123 |
| TCA1 | C1 *^OE^gltA* | 83-127 | 45-67 | 13 | 135-161 |
| TCA5 | C1 *ΔiclR* | 81-103 | 91-116 | 10-22.4 | 103 |
| TCA8 | C1 *^OE^gltA ΔiclR* | 98 | 16-25 | 15-16 | 90-103 |
| GU1 | C1 *ΔptsG* | 44 | 138 | - | 26-58 |
| AP9 | C1 *Δpta ΔpoxB ΔadhE ΔadhP* | 81-94 | 45-64 | 31-77 | 116-155 |
| APTCA2 | C1 *Δpta ΔpoxB ΔiclR* | 78-88 | 40-45 | 20-100 | 97-174 |
