# Supplementary material for: Minimizing acetate formation from overflow metabolism in Escherichia coli: comparison of genetic engineering strategies to improve robustness toward sugar gradients in large-scale fermentation processes
Source: Front Bioeng Biotechnol. 2024 Feb 14;12:1339054. doi: 10.3389/fbioe.2024.1339054 (PMC10899681; doi:10.3389/fbioe.2024.1339054)
Supplement: Supplementary file 4 [file Table2.docx]

**Table S2**. Overview of oligos used in this study

| **Targeted gene** | **ssDNA ID** | **Sequence (5’ to 3’)** |
| --- | --- | --- |
| *adhE* | OL-0461 | ATCACCGCACTGACTATACTCTCGTATTCGAGCAGATGATTTACTAAAAAAGTTTAACATTATCAGGAGAGCATTTCAGTAGCGCTGTCTGGCAACATAAACGGCCCCTTCTGGGCAATGCCGATCAGTTAAGGATTAGTTGACCGATCC |
| *adhP* | OL-0467 | TCCATACTGGGTAGTGGCGAATAAATCTCATTTGCCTCACCTGCTATGCAGAACATCATCCGAAAAGGAGGAACTGAGGCCTTTGCTGCGACTGCCATGTTCGGGTCGCAGCATCGCACACTCTCCAACATGAAATGGCTAAATGGATTA |
| *gltA* | OL-0198 | TGACAATCATTCAACAAAGTTGTTACAAACATTACCAGGAAAAGCATATATGGTTGATTGCTAAGTTGTAAATATTTTAACCCGCCGTTCATATGGCGGG |
| *iclR* | OL-0329 | TCAGTAACTATTGCATTAGCTAACAATAAAAATGAAAATGATTTCCACGATACAGAAAAAAGAGACTGTCCTTTTTCTGGCGGGCAGAGGCAATATTCTGCCCATCATACCTGAGTGGCAATAGAATAAGGGTGTCTGTT |
| *nagE* | OL-0502 | TCGGCAGTACAATTTGCAGCAAAATAAAAATACGGCTTGAAACGAGCCAAATAGGGTTCTCGTAGGGGGAATAAGTCTGCTTTATGCCTGATGCGACGCTTGAGCGTCGCATCCAACAATGACAAGCGGTGGAGATCTTCTCTGCCGCTT |
| *pckA* | OL-0508 | TGAGCCTTGTCGCGGTTAACACCCCCAAAAAGACTTTACTATTCAGGCAATACATATTGGCTAAGGAGCAGTGAATGATTTGAAGCTGGAGAATATCTATCCAGTATCTTATAGAAAGCAAAACGGGAGGCACCTTCGCCTCCCGTTTAT |
| *poxB* | OL-0624 | TTCTCTCCCATCCCTTCCCCCTCCGTCAGATGAACTAAACTTGTTACCGTTATCACATTCAGGAGATGGAGAACCAAAGGGTGGCATTTCCCGTCATAATAAGGACATGCCATGATTGATTTACGCAGTGATACCGTTACCCGACCAAGC |
| *pta* | OL-0515 | GAGAATAAAAAACCGGAAATAGTGATTATTTCCGGTTCAGATATCCGCAGCGCAAAGCTGCGGATGATGACGAGAGGTTTATCCTCTTTCGTTACCGCCGATTTGGCGGGTTACAAAACAGCACCGCCAGCTGAGCTGGCGGTGTGAAAT |
| *ptsG* | OL-0367 | CACGTATCAATTCTGAATAACACCTGTAAAAAAGGCAGCCATCTGGCTGCCTTAGTCTCCCCAACGTCTTACGGAAATTGAGAGTGCTCCTGAGTATGGGTGCTTTTTTTACGTTCTCACGCGTGGCAAGGGGGGAGAGCCTCGCCGTGT |
| **Targeted gene** | **Promoter ID** | **Sequence (5’ to 3’)** |
| *acs, gltA* | Pcon3_70UTR | ATGCGCAAATTTGACGGCTAGCTCAGTCCTAGGTACAGTGCTAGCATGCCTACAAGCATCGTGGAGGTCCGTGACTTTCACGCATACAACAAACATTAACCAAGGAGGAAACAGCT |
| *adhE* | PglpF_SD7 | ATGCGCAAATGCGGCACGCCTTGCAGATTACGGTTTGCCACACTTTTCATCCTTCTCCTGGTGACATAATCCACATCAATCGAAAATGTTAATAAATTTGTTGCGCGAATGATCTAACAAACATGCATCATGTACAATCAGATGGAATAAATGGCGCGATAACGCTCATTTTATGACGAGGCACACACATTTTAAGTTCGATATTTCTCGTTTTTGCTCGTTAACGATAAGTTTACAGCATGCCTACAAGCATCGTGGAGGTCCGTGACTTTCACGCATACAACAAACATTAACCAAGAGCAAAACAGCT |
| *adhP, gltA, ppc* | PglpF | ATGCGCAAATGCGGCACGCCTTGCAGATTACGGTTTGCCACACTTTTCATCCTTCTCCTGGTGACATAATCCACATCAATCGAAAATGTTAATAAATTTGTTGCGCGAATGATCTAACAAACATGCATCATGTACAATCAGATGGAATAAATGGCGCGATAACGCTCATTTTATGACGAGGCACACACATTTTAAGTTCGATATTTCTCGTTTTTGCTCGTTAACGATAAGTTTACAGCATGCCTACAAGCATCGTGGAGGTCCGTGACTTTCACGCATACAACAAACATTAACCAAGGAGGAAACAGCT |
| **Genetic cassette** | **Oligo ID** | **Sequence (5’ to 3’)** |
| *CP6-galK* | O982 | CATGTGGGAGTTTATTCTTGACACAG |
| *CP6-galK* | O983 | TCAGCACTGTCCTGCTCCTTGTGATG |
