# Supplementary material for: Minimizing acetate formation from overflow metabolism in Escherichia coli: comparison of genetic engineering strategies to improve robustness toward sugar gradients in large-scale fermentation processes
Source: Front Bioeng Biotechnol. 2024 Feb 14;12:1339054. doi: 10.3389/fbioe.2024.1339054 (PMC10899681; doi:10.3389/fbioe.2024.1339054)
Supplement: Supplementary file 5 [file DataSheet2.docx]

**
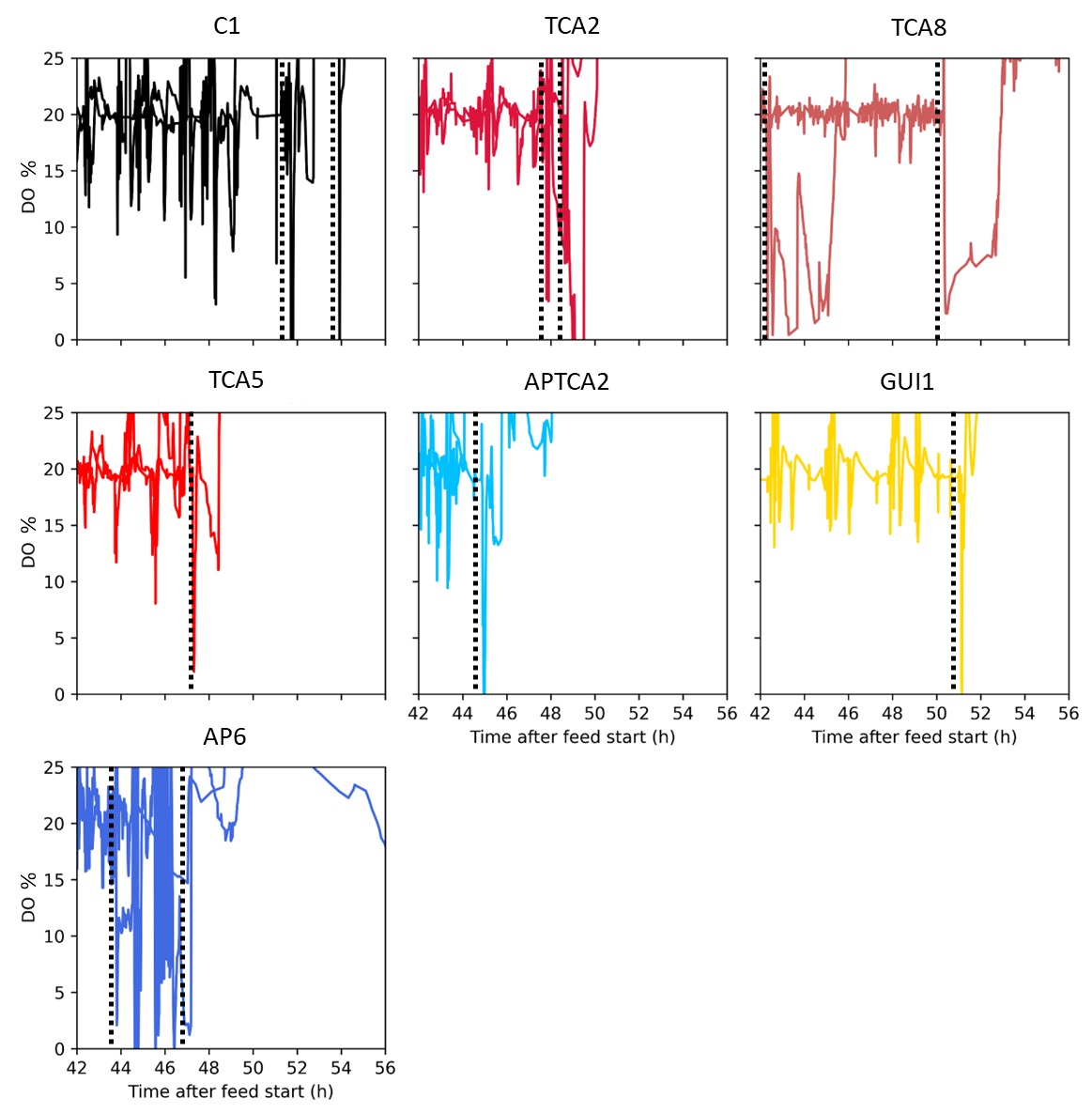
**

**Figure S2.** DO values from fed-batch fermentations after a 10 g/L bolus addition of glucose. The bolus additions are marked with dashed vertical lines. Each subplot shows DO results from two or three replicate fermentations.
