# Supplementary figures and images for: Minimizing acetate formation from overflow metabolism in Escherichia coli: comparison of genetic engineering strategies to improve robustness toward sugar gradients in large-scale fermentation processes
Source: Front Bioeng Biotechnol. 2024 Feb 14;12:1339054. doi: 10.3389/fbioe.2024.1339054 (PMC10899681; doi:10.3389/fbioe.2024.1339054)

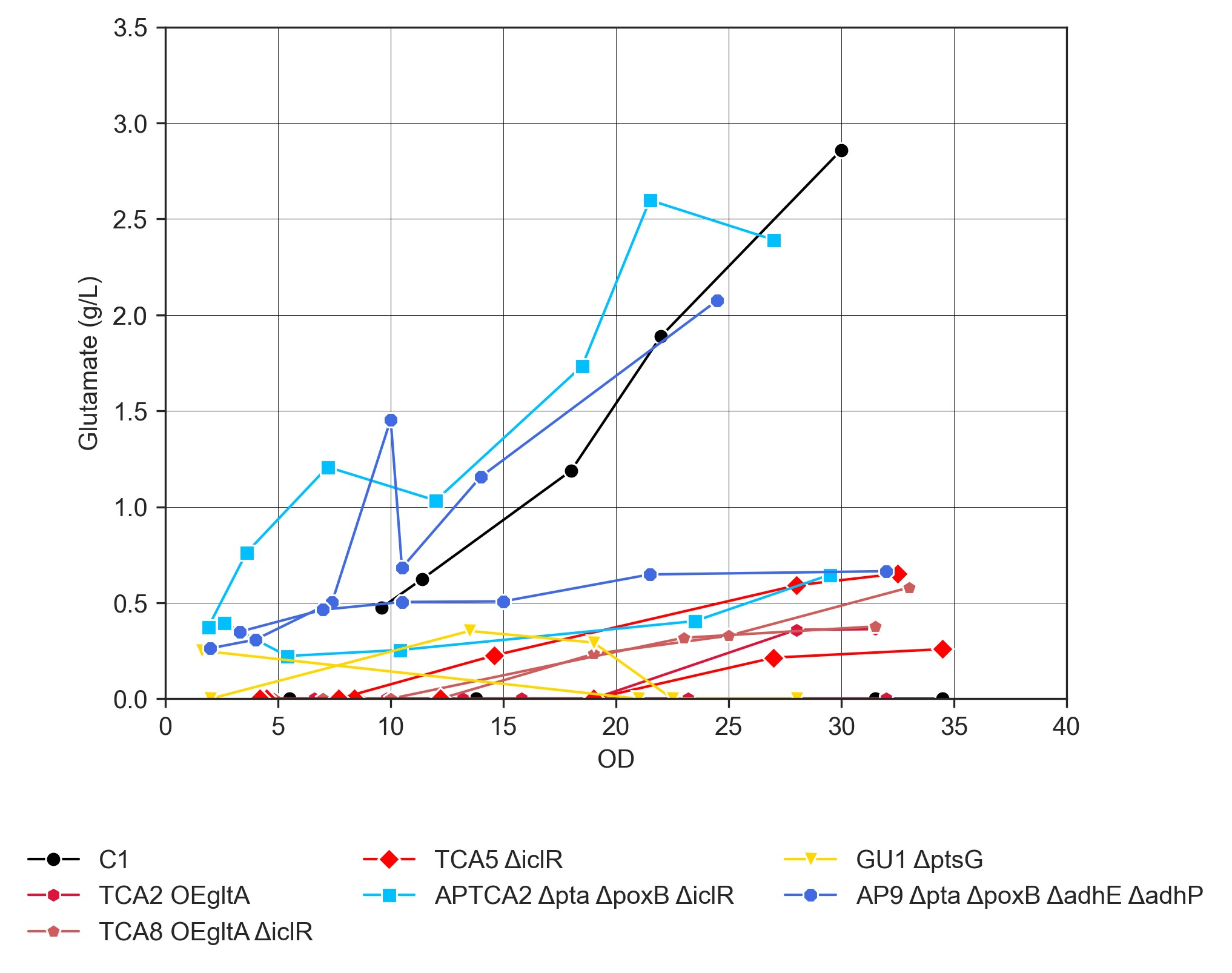


**Figure S1.** Glutamate concentrations in the batch phase in the bioreactor experiments.

Supplement: Supplementary file 6 [file DataSheet1.docx]
